# Design, Synthesis and Biological Evaluation of New Substituted Diquinolinyl-Pyridine Ligands as Anticancer Agents by Targeting G-Quadruplex

**DOI:** 10.3390/molecules23010081

**Published:** 2017-12-30

**Authors:** Rabindra Nath Das, Edith Chevret, Vanessa Desplat, Sandra Rubio, Jean-Louis Mergny, Jean Guillon

**Affiliations:** 1 Université de Bordeaux, ARNA laboratory, INSERM U1212, UMR CNRS 5320, UFR des Sciences Pharmaceutiques, 33076 Bordeaux CEDEX, France; rabindra.das@u-bordeaux.fr (R.N.D.); sandra.rubio@u-bordeaux.fr (S.R.); 2 Université de Bordeaux, INSERM U1053, Cutaneous Lymphoma Oncogenesis Team, 33076 Bordeaux CEDEX, France; edith.chevret@u-bordeaux.fr; 3 Université de Bordeaux, INSERM U1035, Cellules souches hématopoïétiques normales et leucémiques, UFR des Sciences Pharmaceutiques, 33076 Bordeaux CEDEX, France; vanessa.desplat@u-bordeaux.fr; 4 Institute of Biophysics of the CAS, v.v.i., Královopolská 135, 612 65 Brno, Czech Republic

**Keywords:** G-quadruplex, diquinolinyl-pyridine, G4 ligands, FRET-melting, circular dichroism, telomerase, antiproliferative activity, cancer

## Abstract

G-quadruplexes (G4) are stacked non-canonical nucleic acid structures found in specific G-rich DNA or RNA sequences in the human genome. G4 structures are liable for various biological functions; transcription, translation, cell aging as well as diseases such as cancer. These structures are therefore considered as important targets for the development of anticancer agents. Small organic heterocyclic molecules are well known to target and stabilize G4 structures. In this article, we have designed and synthesized 2,6-di-(4-carbamoyl-2-quinolyl)pyridine derivatives and their ability to stabilize G4-structures have been determined through the FRET melting assay. It has been established that these ligands are selective for G4 over duplexes and show a preference for the parallel conformation. Next, telomerase inhibition ability has been assessed using three cell lines (K562, MyLa and MV-4-11) and telomerase activity is no longer detected at 0.1 μM concentration for the most potent ligand **1c**. The most promising G4 ligands were also tested for antiproliferative activity against the two human myeloid leukaemia cell lines, HL60 and K562.

## 1. Introduction

Single stranded guanine-rich nucleic acid sequences may fold into non-canonical four-stranded secondary structures known as G-quadruplexes (G4) [1,2]. These structures are formed through π-π stacking of G-quartets which involve four guanines organized in a plane via eight Hoogsteen type H-bonding [1,2]. G4-structures are stabilized in the presence of monovalent cations such as K^+^ or Na^+^. More than 370,000 sequences have been recognized to form potential G4 structure in the human genomes [3,4] especially G-rich regions of telomeres [5], promoter regions [6,7], introns, immunoglobulin switch regions, DNA replication origins and the untranslated regions of messenger RNAs [8]. G4-structures are mostly involved in regulating various biological processes such as maintenance of genetic information [9], replication [10], transcription [11], cell aging, translation, and diseases such as cancer [12,13]. Thus, G-quadruplexes are considered as an important target for the development of anti-cancer drugs and therapeutic applications [14]. Moreover, down-regulation of transcription has been observed by small molecules that are able to induce and stabilize G4 structures in the promoters of several oncogenes such as *c-myc* [15], *c-kit* [16], KRas [17], and HSP90 [18]. Telomerase, as a ribonucleoprotein reverse transcriptase enzyme, uses its own RNA as a template to synthesize telomere DNA sequences and so elongates telomeres. Further, telomerase acts a key regulator of long-term proliferation [19]. High level of telomerase is observed in most human cancer cells [20]. Both inhibition of telomerase activity through G4 structure stabilization or induction of the DNA damage response through telomere uncapping can prompt a proliferation arrest resulting in anticancer activity. Hence, the small molecules which can target and stabilize G4-structures in human genome could act as potential anticancer agents [14,15,16,17,18,21].

The large planar aromatic surface of a terminal G-quartet provided a rationale for the development of planar G4 ligands such as macrocyclic porphyrin [22,23,24,25] or telomestatin derivatives [26,27,28,29,30], polyaromatic fused molecules which include acridines [31,32,33], phenanthrolines [34,35,36,37], quinolones [38], quinones [39] etc. Based on these chemical structures a number of G4 binding small molecules have been developed during the last two decades [21,22,23,24,25,26,27,28,29,30,31,32,33,34,35,36,37,38,39]. Many of these ligands are selective for G4 structures over duplex DNA, but the design of a ligand specific for a given G4 structure is still challenging.

By taking the challenge to expand the number of effective G4 ligands as potential anticancer agents, herein new diquinolinyl-pyridine derivatives have been designed and synthesized. The ability for G4 thermal stabilization has been screened against the human telomeric quadruplex (F21T) through fluorescence resonance energy transfer (FRET) melting assays [40]. Further, the selectivity over duplex and specificity for different G4 topologies has been investigated. We established that the diquinolinyl-pyridine part offers an extended aromatic surface for π-π stacking interactions with the G-quartets and the amine side chains guidance the stabilizing ability. The most promising G4 ligands were then tested for antiproliferative activity against two human myeloid leukaemia cell lines.

## 2. Results

### 2.1. Design and Synthesis of the Ligands

**Figure 1:**
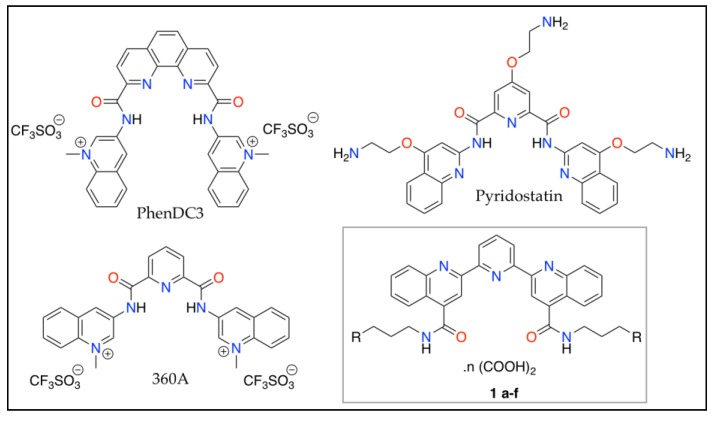
The structures of Phen-DC3, pyridostatin, 360A and newly designed 2,6-di-(4-carbamoyl-2-quinolyl)pyridine derivatives **1a**–**f**.

The quinoline ring is one of the most useful scaffolds found in numerous natural products and pharmaceuticals with an extensive range of biological activities. This framework is also present in many well-known potent G-quadruplex ligands such as Phen-DC3, pyridostatin or 360A [31,32,33,34,35,36,37,38,39]. By considering previously reported G-quadruplex DNA binding ligands with a large flat aromatic surface and positively charged lipophilic lateral chains, herein we have designed pyridine di-quinoline core with the possibility to attach to various carboxamide side chains (Figure 1).

**Scheme 1:**
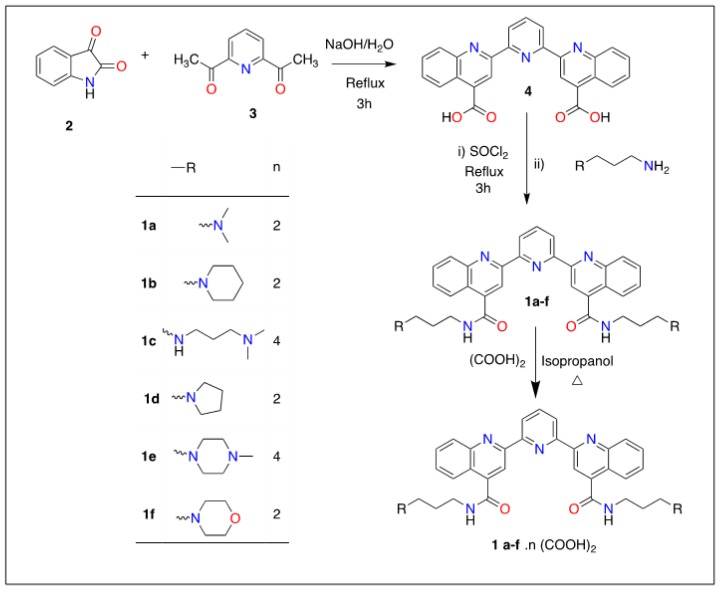
Structure of **1a**–**f** and synthetic scheme for diquinolinyl-pyridine derivatives **1a**–**f**.

The reported 2,6-di-(4-carbamoyl-2-quinolyl)pyridine derivatives **1a**–**f** were synthesized in three steps from the commercially available isatin (**2**) and 2,6-diacetylpyridine (**3**) (Scheme 1). 2,6-Diacetylpyridine reacts with isatin in alkaline condition through double Pfitzinger reaction to give the 2,6-di-(4-carboxamido-2-quinolyl)pyridine **4** after acidification with hydrochloric acid [41]. The diacid chloride was obtained by refluxing the 2,6-di-(4-carboxy-2-quinolyl)pyridine **4** in thionyl chloride. This diacid chloride **3** was then coupled with various propylenamines to afford the 2,6-di-(4-carbamoyl-2-quinolyl)pyridine derivatives **1a**–**f**. These diquinolinylpyridine compounds **1a**–**f** were then converted into their ammonium oxalate salts **1a**–**f** by treatment with oxalic acid in refluxing isopropanol (Table 1).

### 2.2. Screening of the Ligands

The ability of the diquinolinyl-pyridine derivatives **1a**–**f** to bind and stabilize G4 structures was tested by the FRET melting assay [38] against the fluorescently labelled human telomeric quadruplex (F21T; 0.2 µM), with different ligand concentrations ranging from 0.5 to 10 µM in K^+^ and in Na^+^ buffer conditions. This experiment provides an estimation of the G4 stabilization effects of the ligands as changes in melting temperature (∆T_m_). This stabilization (∆T_m_) is the difference between the half denaturation (T_1/2_) temperature of F21T in the presence or absence of the ligand. As expected, the stabilization effects are dependent on ligand concentration: the ∆T_m_ values at 5 µM ligand are higher than that at 2 µM (Table 2, Supplementary Materials Figures S1 and S2). All the newly synthesized compounds except **1f** are able to stabilize the G4 structures and the stabilization ability is more prominent in K^+^ than in Na^+^. The ∆T_m_ of F21T for the ligands **1a**–**e** ranged from 6.8 to 18.7 °C at 2 µM and from 12.8 to 23.5 °C at 5 µM in K^+^ buffer. In Na^+^ containing buffer, the stabilization ranged from 2.6 to 15.5 °C at 5 µM for the ligands **1a**–**e**. The in vitro topology of the telomeric G4-oligonucleotides depends on various factors such as sequence, loop length and buffer conditions. In K^+^ buffer, F21T adopts a mixture of antiparallel and 3 + 1 hybrid conformations while in Na^+^ it predominantly adopts an antiparallel G4 conformation. Hence, cation dependent FRET melting assays revealed that the ligands are comparatively less effective on the antiparallel G4 conformation. The ligand **1f** with 3-morpholinopropyl substituents has been observed to have less stabilizing ability for F21T sequence. Based on these results, compounds **1a**–**d** were chosen for further biophysical studies.

**Figure 2:**
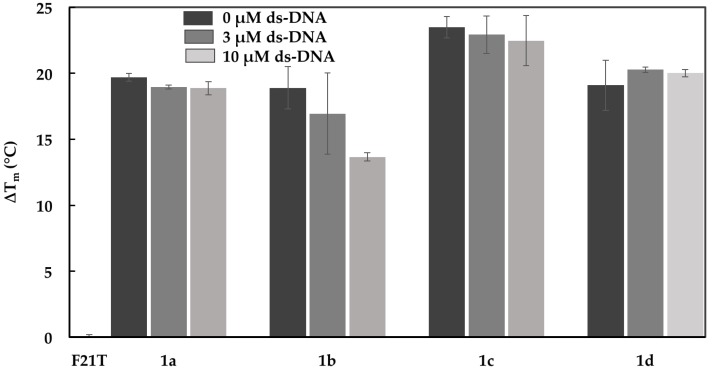
Stabilization of F21T (0.2 µM) by **1a**–**d** (5 µM) in the presence of various concentrations of a double-stranded competitor, ds26. Comparison of ∆T_m_ in K^+^ conditions in the absence of competitor (black) vs. ∆T_m_ in K^+^ conditions in the presence of ds26 (grey bars). Error bars correspond to SD of at least three independent experiments. Experiments were performed in 10 mM lithium cacodylate buffer (pH 7.2), 90 mM LiCl, and 10 mM KCl. T_m_ in absence of ligands of F21T is 52.4 ± 0.3 °C.

To probe the G4 selectivity of the ligands **1a**–**d** over duplex DNA, competitive FRET melting assays were performed in presence of large excess of a non-fluorescent self-complementary double-stranded DNA (ds26) competitor. For this experiment, 5 µM ligand concentration was used for stabilization of the human telomeric F21T (0.2 µM) in the presence of a 15 to 50-fold excess of a double-stranded DNA competitor (Figure 2). The results showed that the stabilizing abilities of the ligands **1a**, **c**, **d** for G4 conformations are nearly unaffected in presence of ds-DNA competitor. For ligand **1b** the decrease in F21T thermal stability is observed in the presence of a large excess of ds26 which indicates a decent selectivity for **1b** towards the G4 structure.

Ligands **1a**–**d** were also tested for their ability to stabilize other G4-forming sequences which are known to form various G4 topologies. For this experiment, nine different fluorescently labelled G4 forming sequences were chosen; parallel G4 forming sequences such as F25cebT [42], extracted from human minisatellite repeats, FmycT [43] and Fkit1T [44], extracted from human oncogene promoter; antiparallel G4 topologies such as FtbaT [45], thrombin binding aptamer sequence, Fbom17T [43], obtained from the *Bombyx mori* telomeres and F21ctaT [46], a mutant variant derived from the DNA human telomeric sequence; *Plasmodium* telomeric G4 sequences FPf1T and FPf8T extracted from *Plasmodium falciparum* [47]. Finally, the fluorescently labelled oligonucleotide FdxT was used as a non-G4-forming control sequence, as it folds into an intramolecular duplex.

**Figure 3:**
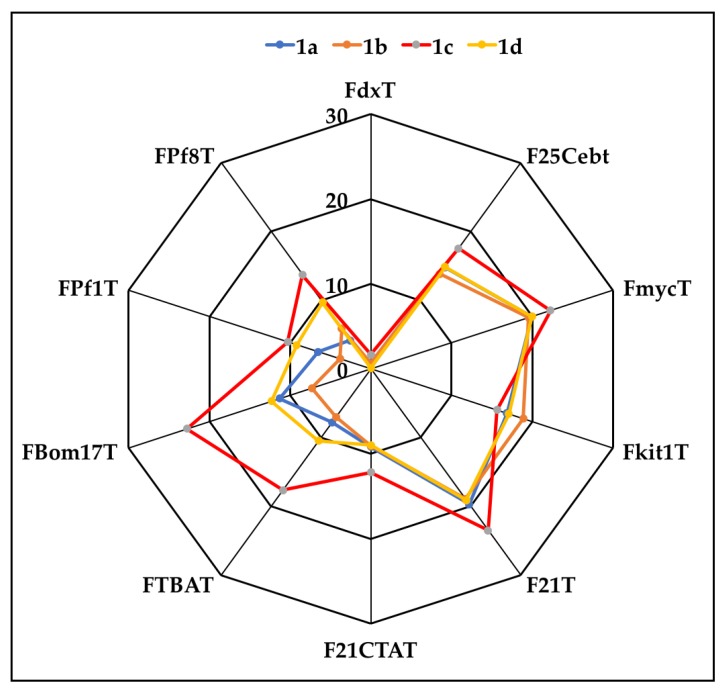
Stabilization specificity profile of **1a**–**d** (5 µM) toward various G4 oligonucleotides. The difference in T_m_ in presence and absence of **1a**–**d**, ∆T_m_, in °C is plotted for each sequence. 9 quadruplexes and one duplex (FdxT) were tested. Experiments were performed in 10 mM lithium cacodylate buffer (pH 7.2), 90 mM LiCl, and 10 mM KCl. T_m_ in absence of ligands: FdxT (T_m_ = 66.6 ± 0.7 °C), F25cebT (T_m_ = 68.3 ± 0.7 °C); FmycT (T_m_ = 64.8 ± 0.3 °C); Fkit1T (T_m_ = 66.5 ± 0.3 °C ); F21T (T_m_ = 52.4 ± 0.3 °C); F21ctaT (T_m_ = 56.6 ± 0.2 °C); FtbaT (T_m_ = 46.1 ± 0.3 °C); Fbom17T (T_m_ = 44.7 ± 0.6 °C); FPf1T (T_m_ = 45.8 ± 0.9 °C); FPf8T (T_m_ = 45.7 ± 0.2 °C).

The radar plot shown in Figure 3 and Table 3 show that compounds **1a**–**d** are able to stabilize all the G4 forming sequences.

Compounds **1a**, **1b** and **1d** are more specific for the parallel topologies (14.8–19.9 °C for **1a**; 13.8–19.5 °C for **1b** and 14.9 to 19.9 °C for **1d**) than for antiparallel G4 structures (7.8–11.2 °C for **1a**; 6.9–9.0 °C for **1b**; 8.9–12.3 °C for **1d**) which is in consistent with the results obtained from FRET assay with F21T in Na^+^ condition. Compounds **1a**, **1b** and **1d** have higher affinities towards the *c-myc* and *c-kit1* human oncogene promoter sequences suggesting their ability to inhibit *c-myc* and *c-kit* expression in cancer cells.

### 2.3. CD Experiments

**Figure 4:**
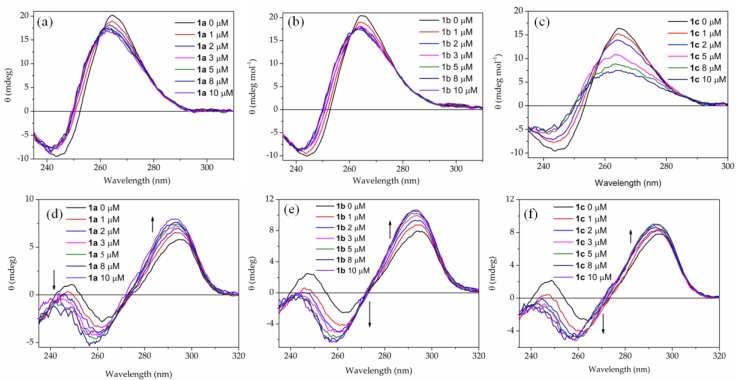
CD titration of c-myc (2 μM) with (**a**) **1a**, (**b**) **1b**, (**c**) **1c** and 22CTA (2 μM) with (**d**) **1a**, (**e**) **1b**, (**f**) **1c**. CD spectra were recorded in 10 mM lithium cacodylate buffer (pH 7.3), 90 mM LiCl, and 10 mM KCl.

Circular dichroism (CD) spectroscopy was employed to investigate the effect of the ligands on the conformation of the G-quadruplexes upon binding (Figure 4). As obtained from previous experiments (Figure 3), ligands **1a** and **1b** have higher affinities towards the parallel G-quadruplex over antiparallel structures, the effect of these ligands towards the conformation of two different unlabeled sequences, *c-myc* (TTGAG_3_TG_3_TAG_3_TG_3_TAA) and 22CTA (AG_3_CTAG_3_CTAG_3_CTAG_3_) were investigated through CD titrations experiment. The CD spectrum of c-myc associated with a negative peak at 244 nm and a positive peak at 265 nm in 10 mM KCl buffer condition is indicative of a parallel conformation (Figure 4a–c).

Upon addition of ligands **1a** and **1b** to the solution of c-myc, a slight decrease of elipticity at both points (244 and 265 nm) were observed without disturbing the shape of the CD spectra while for **1c** the decrease of ellipticity was more pronounced. The CD spectrum of 22CTA is associated with a positive peak at 295 nm and a negative peak at 265 nm, indicative of an antiparallel conformation (Figure 4d–f). Upon addition of ligands **1a** and **1b** to 22CTA the increment in elipticity at both points were observed without altering the shape of spectra.

### 2.4. Detection of Telomerase Activity in Cell Lysates

**Figure 5:**
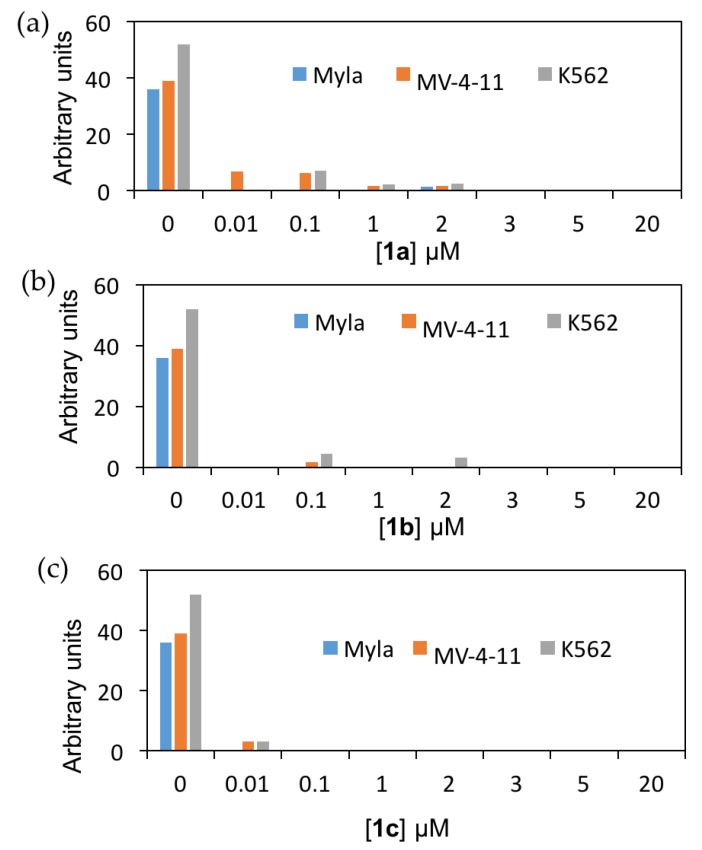
Telomerase activity (TA) in My-La, MV-4-11 and K562 cell lysates was investigated in the presence of 0–20 µM of (**a**) **1a**, (**b**) **1b** and (**c**) **1c** ligands.

As the human telomeric motif F21T was among the most stabilized G4 (Figure 3) sequences, we investigated whether compounds **1a**–**c** may interfere with telomeric functions. We first assayed their effects on telomerase activity in cell lysates. Telomerase activity in K562, MV-4-11 and My-La cell lysates was investigated in the presence of 0–20 µM of the ligands (Figure 5a–c). As expected at 0 µM telomerase activity was detectable in K562, MV-4-11 and My-La cell lines, as expected. All the three compounds abolished telomerase activity at 5 and 20 µM. At 2 µM concentration of **1a** telomerase activity was still observed for all cell lines tested (Figure 5a), while in the presence of **1c** telomerase activity was no longer detected for all cell lines (Figure 5c). At 2 µM, diquinolinylpyridine **1b** abolished telomerase activity for two (My-La and MV-4-11) of the three cell lines (Figure 5b). Next, K562 and MV-4-11 cells lysates were investigated for telomerase activity in presence of <2 µM concentration of **1b** and **1c**. For both cell lines at 1 µM of **1b** and **1c** no telomerase activity was detected. At 0.1 µM of **1b,** telomerase was still active in both cell lines, however **1c** is able to inhibit telomerase activity at the same concentration of 0.1 µM. While tested at 0.01 µM of **1c**, telomerase activity for both cell lines was still present.

### 2.5. Cytotoxic Effects on Human Cells

The most promising G4 ligands **1a**, **1b** and **1c** were then tested for antiproliferative activity against two human myeloid leukaemia cell lines, HL60 and K562 (Table 4). **1b** was found to be the most active compound against the HL60 human acute myeloblastic leukemia cell line, with a IC_50_ of 18 µM, whereas **1a** and **1c** were found inactive (IC_50_ > 50 µM). Diquinolinylpyridine **1b** also inhibited the growth of K562 human myeloid leukaemia cells with an IC_50_ value of 3 µM. The two other 2,6-di-(4-carbamoyl-2-quinolyl)pyridine derivatives **1a** and **1c** were also found inactive against this cell line K562 (>50 µM). In addition, diquinolinylpyridines **1a**–**c** were tested on activated (PBMNC + PHA) human peripheral blood mononuclear cells to evaluate their cytotoxicity on normal cells. **1a** and **1c** presented an IC_50_ superior to 50 μM against lymphocytes. These preliminary results could be used to determine their respective range of toxic concentration. Indexes of selectivity (IS) were defined as the ratio of the IC_50_ value on the human mononuclear cells to the IC_50_ value on the K562, and HL60 lines. This just led to identify compound **1b** with index of selectivity 1.7 on the human myeloid leukemic cell lines K562.

## 3. Experimental Section

### 3.1. General Information

Commercially available reagents were used as received without additional purification. Melting points were determined with an SM-LUX-POL Leitz hot-stage microscope (Leitz GMBH, Midland, ON, Canada) and are uncorrected. IR spectra were recorded on a Nicolet 380FT-IR spectrophotometer (Thermo Electron Scientific Instruments LLC, Madison, WI, USA). NMR spectra were recorded with tetramethylsilane as an internal standard using an AVANCE 300 spectrometer (Bruker BioSpin, Wissembourg, France). Splitting patterns have been reported as follows: s = singlet; bs = broad singlet; d = doublet; t = triplet; q = quartet; dd = double doublet; ddd = double double doublet; dt = double triplet; m = multiplet. Analytical TLC were carried out on 0.25 precoated silica gel plates (POLYGRAM SIL G/UV254, Fisher Scientific SAS, Illkirch, France) and visualization of compounds after UV light irradiation. Silica gel 60 (70–230 mesh) was used for column chromatography. Mass spectra were recorded on an Ultraflex III TOF/TOF system (Bruker Daltonics, Fällanden, Switzerland), equipped with a 200 Hz smartbeam laser (355 nm) and operating in reflectron positive ion mode. Mass spectra were acquired over the *m*/*z* range 300–5000 by accumulating data from 1000 laser shots for each spectrum. The instrumental conditions employed to analyse molecular species were the following: ion source 1: 25.08 kV; ion source 2: 21.98 kV, lens: 11.03 kV, pulsed ion extraction: 30 ns, reflector: 26.39 kV, reflector 2: 13.79 kV. Matrix suppression was activated by deflection mode: suppression up to 450 Da. Mass calibration was performed for each sample with a peptide calibration mixture (8,206,195, Peptide Calibration Standard, Bruker Daltonics). The instrument was controlled using Bruker’s flexControl 3.4 software, and mass spectra were analysed in Bruker’s FlexAnalysis 3.4 software. Elemental analyses were found within ±0.4% of the theoretical values.

### 3.2. Chemistry

#### 3.2.1. Synthesis of 2,2′-(pyridine-2,6-diyl)*bis*(quinoline-4-carboxylic acid) (**4**)

Isatin (**2**) was added in NaOH (33%) solution under ice-cold condition and stirred for 10 min to dissolve. 2,6-diacetylpyridine (**3**) was added portion wise to the mixture and it was allowed to warm to 60 °C for 30 min to get a red precipitate. The reaction mixture was cooled and the precipitate was filtered, washed with minimum amount of cold water and then with ether (3 times). After drying the precipitate was dissolved in hot 8 mL water and 1 N HCl was used to adjust the pH to 5. The red precipitate was filtered and washed with water and then petroleum ether for drying to get the pure product **4**.

#### 3.2.2. General Procedure for the Synthesis of **1a**–**f**

To diacid **4** (200 mg) SOCl_2_ (5 mL) was added and refluxed for 2 h. Excess SOCl_2_ was evaporated and dried. Then the amine was added directly in excess amount (3 mL) to diacyl chloride under cold condition. The reaction mixture was stirred at room temperature for 24 h. It was cooled and 10 mL of water was added and stirred for 10 mins. If the precipitate appeared, then it was filtered and washed with water and petroleum ether to dry. If precipitate did not appear, then it was extracted with dichloromethane and the organic layer was evaporated to get the final product.

*2,2′-(Pyridine-2,6-diyl)bis(N-(3-(dimethylamino)propyl)quinoline-4-carboxamide)* (**1a**). Beige crystals, Yield: 91%, m.p. = 162–164 °C; ^1^H-NMR δ (CDCl_3_ and 10% MeOD) 8.724 (s, 2H, –CH), 8.649 (d, 4H, *J* = 7.50 Hz, –CH), 8.218 (d, 2H, *J* = 8.70 Hz, –CH), 8.074 (d, 2H, *J* = 8.70 Hz, –CH), 7.966 (t, 1H, *J* = 7.50 Hz, –CH), 7.667 (m, 2H, –CH), 7.485 (m, 2H, –CH), 3.573 (t, 4H, *J* = 6.9 Hz, –CH_2_), 2.402 (t, 4H, *J* = 6.9 Hz, –CH_2_), 2.205 (s, 12H, –CH_3_), 1.769 (qn, 4H, *J* = 6.9 Hz, –CH_2_). ^13^C-NMR δ (CDCl_3_) 169.090, 156.716, 156.411, 149.643, 144.570, 139.378, 131.308, 131.212, 128.952, 126.923, 125.874, 123.619, 117.804, 59.203, 46.277, 41.094, 27.244. MALDI-TOF MS *m*/*z* [M + H]^+^ Calcd. C_35_H_40_N_7_O_2_: 590.3243, Found: 590.322.

*2,2′-(Pyridine-2,6-diyl)bis(N-(3-(piperidin-1-yl)propyl)quinoline-4-carboxamide)* (**1b**). Beige crystals, Yield: 92%, m.p. = 158–160 °C; ^1^H-NMR δ (CDCl_3_) 9.293 (br, 2H, –NH), 8.906 (s, 2H, –CH), 8.819 (d, 2H, *J* = 7.8 Hz, –CH), 8.382 (d, 2H, *J*= 8.1 Hz, –CH), 8.252 (dd, *J* = 8.1 and 1.2 Hz, –CH), 8.112 (t, 1H, *J* = 7.8 Hz, –CH), 7.838–7.783 (ddd, 2H, *J* = 8.1, 6.9 and 1.2 Hz, –CH), 7.678–7.623 (ddd, 2H, *J* = 8.1, 6.9 and 1.2 Hz, –CH), 3.783 (t, 4H, *J* = 5.4 Hz, –CH_2_ ), 2.557 (t, 4H, *J* = 5.4 Hz, –CH_2_), 2.304 (br, 8H, –CH_2_), 1.877 (qn, 4H, *J* = 5.4 Hz, –CH_2_), 1.000 (br, 12H, –CH_2_). ^13^C-NMR δ (CDCl_3_) 169.460, 156.942, 156.332, 149.706, 144.456, 139.529, 131.530, 131.325, 129.179, 126.715, 126.005, 123.727, 118.155, 58.925, 55.584, 41.038, 26.470, 25.826, 24.930. MALDI-TOF MS *m*/*z* [M + H]^+^ Calcd. for C_41_H_48_N_7_O_2_: 670.3869, Found: 670.382.

*2,2′-(Pyridine-2,6-diyl)bis(N-(3-((3-(dimethylamino)propyl)amino)propyl)quinoline-4-carboxamide)* (**1c**). Beige crystals, Yield: 80%, m.p. = 148–150 °C; ^1^H-NMR δ (CDCl_3_) 8.941 (s, 2H, –CH), 8.912 (br, 4H, –NH), 8.783 (d, 2H, *J* = 7.8 Hz, –CH), 8.407 (d, 2H, *J* = 8.4, –CH), 8.242 (d, 2H, *J* = 8.4 Hz, –CH), 8.099 (t, 1H, *J* = 7.8 Hz, –CH), 7.824–7.776 (m, 2H, –CH), 7.667–7.619 (m, 2H), 3.773 (t, 4H, *J* = 5.7 Hz, –CH_2_), 2.902 (t, 4H, *J* = 5.7 Hz, –CH_2_), 2.556 (t, 4H, *J* = 6.9 Hz, –CH_2_), 1.993–1.952 (m, 8H, –CH_2_), 1.840 (s, 12H, –CH_3_), 1.359 (qn, 4H, *J* = 6.9 Hz, –CH_2_). ^13^C-NMR δ (CDCl_3_) 169.028, 156.937, 156.347, 149.805, 144.674, 139.426, 131.692, 131.419, 129.192, 126.450, 125.874, 123.738, 117.926, 59.829, 48.215, 47.235, 46.625, 46.102, 29.364, 26.943. MALDI-TOF MS *m*/*z* [M + H]^+^ Calcd. for C_41_H_54_N_9_O_2_: 704.4400, Found: 704.442.

*2,2′-(Pyridine-2,6-diyl)bis(N-(3-(pyrrolidin-1-yl)propyl)quinoline-4-carboxamide)* (**1d**). Beige crystals, Yield: 94%, m.p. = 156–158 °C; ^1^H-NMR δ (CDCl_3_ and 10% MeOD) 8.815 (s, 2H, –CH), 8.724 (d, 2H, *J* = 7.80 Hz, –CH), 8.304 (dd, 4H, *J* = 7.80 and *J* = 6.90 Hz, –CH), 8.166 (t, 1H, *J* = 7.80 Hz, –CH), 7.840 (m, 2H, –CH), 7.680 (m, 2H, –CH), 3.627 (t, 4H, *J* = 6.90 Hz, –CH), 2,776 (t, 4H, *J* = 8.10 Hz, –CH_2_), 2.750–2.706 (m, 8H, –CH_2_), 1.989 (qn, 4H, *J* = 7,20 Hz, –CH_2_), 1.797–1.786 (m, 8H, –CH_2_). ^13^C-NMR δ (CDCl_3_) 170.070, 157.021, 156.489, 149.602, 144.515, 139.587, 131.608, 131.122, 129.214, 126.424, 125.867, 123.811, 118.141, 58.272, 39.819, 28.851, 24.336. MALDI-TOF MS *m*/*z* [M + H]^+^ Calcd. for C_39_H_44_N_7_O_2_: 642.3556, Found: 642.352.

*2,2′-(Pyridine-2,6-diyl)bis(N-(3-(4-methylpiperazin-1-yl)propyl)quinoline-4-carboxamide)* (**1e**). Beige crystals, Yield: 92%, m.p. = 168–170 °C; ^1^H-NMR δ (CDCl_3_) 9.110, (s, 2H, –NH) 8.894 (s, 2H, –CH), 8.823 (d, 2H, *J* = 7.80 Hz, –CH), 8.394 (d, 4H, *J* = 8.4, –CH), 8.270 (d, 2H, *J* = 8.4 Hz, –CH), 8.121 (t, 1H, *J* = 8.1 Hz, –CH), 7.847–7.800 (m, 2H, –CH), 7.684–7.634 (m, 2H, –CH), 3.782 (t, 4H, *J* = 6.00 Hz, –CH_2_), 2.619 (t, 4H, *J* = 6.0 Hz, –CH_2_), 2.473–2.321 (m, 16H, –CH_2_), 1.885 (t, 8H, *J* = 6.0 Hz, –CH_2_), 1.698 (s, 6H, –CH_3_). ^13^C–NMR δ (CDCl_3_) 168.852, 156.773, 156.298, 149.899, 144.931, 139.549, 131.581, 131.572, 129.183, 126.917, 126.132, 123.721, 117.953, 59.538, 56.063, 54.152, 46.638, 42.729, 25.147. MALDI-TOF MS *m*/*z* [M + H]^+^ Calcd. for C_41_H_50_N_9_O_2_: 700.4087, Found: 700.398.

*2,2′-(Pyridine-2,6-diyl)bis(N-(3-morpholinopropyl)quinoline-4-carboxamide)* (**1f**). Beige crystals, Yield: 90%, m.p. = 156–168 °C; ^1^H-NMR δ (CDCl_3_) 8.863 (s, 2H, –CH), 8.826 (d, 2H, *J* = 7.8 Hz, –CH), 8.731 (br, 2H, –NH), 8.334 (d, 2H, *J* = 8.1, –CH), 8.263 (d, *J* = 8.1 Hz, –CH), 8.126 (t, 1H, *J* = 7.8 Hz, –CH), 7.817 (m, 2H, –CH), 7.641 (m, 2H), 3.784 (t, 4H, *J* = 6.00 Hz ), 3.220 (m, 8H, –CH_2_), 2.655 (t, 4H, *J* = 6.00 Hz, –CH2), 2.445 (m, 8H, –CH_2_), 1.955 (qn, 4H, *J* = 6.00 Hz, –CH_2_). ^13^C-NMR δ (CDCl_3_) 169.018, 156.837, 156.147, 149.865, 144.754, 139.606, 131.762, 131.539, 129.142, 126.650, 125.944, 123.838, 117.626, 67.829, 59.866, 54.815, 42.206, 25.102. MALDI-TOF MS *m*/*z* [M + H]^+^ Calcd. for C_39_H_44_N_7_O_4_ : 674.3455, Found: 674.342.

#### 3.2.3. General Procedure for the Synthesis of Ammonium Salts **1a**–**f**

To a solution of compounds **1a**–**f** (0.4 mmol) in isopropanol (12 mL) was added oxalic acid (8 or 4 mmol). The reaction mixture was heated under reflux for 30 min. The precipitate was filtered, washed with isopropanol then with diethyl ether, and dried under reduced pressure to give the ammonium salts of **1a**–**f**.

### 3.3. Biophysical Assays

#### FRET Melting Assay

The FRET melting assay is based on the fluorescence resonance energy transfer (FRET) phenomenon between two dyes (here: FAM and TAMRA) attached at both extremities of a DNA oligonucleotide. When the oligonucleotides are folded at low temperature the fluorescence emission of the donor is quenched due to the proximity of the acceptor, leading to efficient FRET. Fluorescence emission recovery occurs when temperature is increased due to thermal denaturation of the structure. Fluorescence recording was performed in a Strategene MX3005P real-time PCR device (Agilent Technologies, Santa Clara, CA, USA) at temperatures from 25 to 90 °C using a 492-nm excitation wavelength and a 516-nm detection wavelength. Experiments were performed in 96-well plates, and each condition was tested in duplicate. In each well, 200 nM of labelled oligonucleotide was melted in the presence (or not) of the ligand with (or without) the competitor at concentrations specified.

To determine whether the ligands stabilized the G-quadruplex conformations, we determined the temperature of half denaturation of the G4 in the absence and in the presence of increasing ligand concentrations. For this experiment we used F21T. The oligonucleotide was prefolded in 10 mM lithium cacodylate buffer (pH 7.2), with either 10 mM KCl and 90 mM LiCl (K^+^ condition) or 100 mM NaCl (Na^+^ condition) as previously described [40]. The salt conditions of the experiment were similar to the one used for the G4 prefolding. In addition to the labelled oligonucleotide, the wells contained 0, 1, 2, or 5 µM ligand. To determine ligand G4 vs. duplex selectivity, an unlabelled DNA competitor ds26 was used. The G4 versus duplex selectivity experiments were performed in K^+^ conditions using F21T. Wells contained F21T, 1 µM ligand, and 0, 3, or 10 µM unlabelled competitor ds26. Finally, in order to identify a putative preferred G4 conformation targeted by a ligand, a set of oligonucleotides (Table 5) covering a range of possible G4 conformations was used [42,43,44,45,46,47,48].

### 3.4. Biology

#### 3.4.1. Telomerase Assays

Telomerase activity was assessed using the TRAP assay kit ((TRAPeze^®^ RT telomerase detection kit, Chemicon, Millipore Sigma S7700, Merck KGaA, Darmstadt, Germany) according to manufacturer’s instructions with some modifications. Briefly, 10^6^ cells (MyLa, K562 or MV-4-11) were resuspended in CHAPS lysis buffer and proteins were extracted. Protein extracts were used to extend a synthetic telomeric DNA by PCR amplification (1 cycle of 30 °C for 30 min, telomere template extension with hotstart DNA polymerase activation, followed by a telomeric PCR amplification: 95 °C for 3 min, 2 cycles of 95 °C for 20 s and 49 °C for 20 s, followed by 30 cycles 95 °C for 20 s and 60 °C for 20 s with signal acquisition) in a Stratagene Mx3005P system using specific telomeric PCR quantitative primers [49]. Each sample was measured in duplicate with a control DNA.

#### 3.4.2. Cell Culture

The human leukemic cell lines K562, MV-411 and HL60 were grown in RPMI 1640 medium (Life Technology, Merck KGaA, Darmstadt, Germany) supplemented with 10% fetal calf serum (FCS), antibiotics (100 U/mL penicillin, 100 µg/mL streptomycin) and l-glutamin, (Eurobio, Les Ulis, France) at 37 °C, 5% CO_2_ in air. The toxicity of the selected G4-ligands was also evaluated on non-activated, freshly isolated normal human peripheral blood mononuclear cells (PBMNC), as well as phytohemagglutinin (T lymphoproliferative agent) (PHA)-induced cells. PBMNC from blood of healthy volunteers were obtained following centrifugation on Ficoll gradient. Cells were then incubated in medium alone or induced to enter cell cycle by the addition of PHA (5 µg/mL, Murex Biotech Limited, Dartford, UK). My-La (a gift from Dr. Kaltoft, Institute of Human Genetics, Aarhus University, Denmark) is an aggressive cutaneous T cell lymphoma cell line. My-La cells were grown at 37 °C in a 5% CO_2_ in RPMI media containing 10% fetal bovine serum (FBS). All cell lines were regularly tested for the absence of mycoplasma contamination.

#### 3.4.3. Cytotoxicity Test

The MTS cell proliferation assay (Promega, Madison, WI, USA) is a colorimetric assay that measures the reduction of the tetrazolium MTS into formazan by the mitochondria of viable cells. Cells were washed twice in PBS and plated in quadruplicate into microtiter plate wells in 100 μL culture media with or without G4 ligand at concentrations of 1, 5, 10, 20, and 50 μM for 3 days. After 3 h of incubation at 37 °C with 20 μL MTS per well, the plates were read using an ELISA microplate reader (Thermo, Electrocorporation, Waltham, MA, USA) at 490 nm. The amount of colour produced was directly proportional to the number of viable cells. The results are expressed as the concentrations inhibiting cell growth by 50% after a 3 days incubation period. The IC_50_ was determined by linear regression analysis (Microsoft Excel) and is expressed in μM ± SD.

## 4. Conclusions

We have designed and synthesized new diquinolinyl-pyridines attached to various carboxamide side chains. The compounds **1a**–**e** stabilized the G-quadruplex structure formed by the fluorescently labeled human telomeric DNA sequence F21T. It had also been established that the ligands are selective for G4 over duplexes and show a preference for parallel conformation. The inhibition for telomerase activity by the ligands had been assessed through TRAP assay and the result showed complete inhibition at 0.1 μM concentration for the ligand **1c**. The most promising G4 ligands **1** were also tested for antiproliferative activity against the two human myeloid leukaemia cell lines, HL60 and K562. Thus, the diquinolinyl-pyridine **1b** was determined as the most active compound against the K562 human myeloid leukaemia cells with an IC_50_ value of 3 µM; and with a IC_50_ of 18 µM, against the HL60 leukemia cell line.

## Figures and Tables

**Table 1:** Physical properties of ammonium salts **1a**–**f**.

Compound ^a^	Salt ^b,c^	Elemental Analyses	Feature	Yield (%) ^d^
**1a**	2(COOH)_2_	Anal. Calcd. for C_39_H_43_N_7_O_10_: C, 60.85; H, 5.63; N, 12.74. Found: C, 60.94; H, 5.54; N, 12.86	Yellow powder	81
**1b**	2(COOH)_2_	Anal. Calcd. for C_45_H_51_N_7_O_10_: C, 63.59; H, 6.05; N, 11.54. Found: C, 63.67; H, 5.91; N, 11.43	Yellow powder	84
**1c**	4(COOH)_2_	Anal. Calcd. for C_49_H_61_N_9_O_18_: C, 55.31; H, 5.78; N, 11.85. Found: C, 55.20; H, 5.94; N, 11.74	Brown powder	75
**1d**	2(COOH)_2_	Anal. Calcd. for C_43_H_47_N_7_O_10_: C, 62.84; H, 5.76; N, 11.93. Found: C, 62.95; H, 5.66; N, 11.98	Yellow powder	78
**1e**	4(COOH)_2_	Anal. Calcd. for C_43_H_47_N_7_O_10_: C, 62.84; H, 5.76; N, 11.93. Found: C, 62.95; H, 5.66; N, 11.98	Yellow powder	80
**1f**	2 (COOH)_2_	Anal. Calcd. for C_43_H_47_N_7_O_12_: C, 60.49; H, 5.55; N, 11.48. Found: C, 60.64; H, 5.42; N, 11.67	Yellow powder	82

^a^ Crystallization solvent: iPrOH–H_2_O; ^b^ The stoichiometry and composition of the salts were determined by elemental analyses and obtained values were within ±0.4% of the theoretical values; ^c^ Melting point of the salts were found to be >260 °C; ^d^ The yields only included the conversions into the ammonium oxalates.

**Table 2:** FRET-melting values for compounds **1a**–**f** with F21T in K^+^ or Na^+^ conditions.

Compound	∆T_m_ (°C) ^a^ in K^+^ Condition		∆T_m_ (°C) ^b^ in Na^+^ Condition	
	2 µM	5 µM	2 µM	5 µM
**1a**	15.5 ± 2.1	19.7 ± 0.3	4.7 ± 0.4	7.7 ± 1.0
**1b**	13.8 ± 0.5	18.9 ± 1.6	5.1 ± 0.2	7.8 ± 0.7
**1c**	18.7 ± 1.1	23.5 ± 0.8	10.9 ± 0.1	15.5 ± 1.4
**1d**	13.5 ± 0.5	19.1 ± 1.9	4.1 ± 0.9	6.2 ± 0.9
**1e**	6.8 ± 1.5	12.8 ± 1.6	2.9 ± 1.1	2.6 ± 0.8
**1f**	1.3 ± 0.4	1.9 ± 0.8	0.6 ± 0.2	0.4 ± 0.1

^a^ ∆T_m_ of F21T (0.2 µM) is recorded in 10 mM lithium cacodylate (pH 7.2), 10 mM KCl, and 90 mM LiCl. F21T T_m_ in K^+^ = 52.4 ± 0.3 °C; ^b^ ∆T_m_ of F21T (0.2 µM) is recorded in 10 mM lithium cacodylate (pH 7.2) and 100 mM NaCl. F21T T_m_ in Na^+^ is 48.8 ± 0.9 °C. Error margins correspond to SD of at least three independent experiments.

**Table 3:** Stabilization profile of **1a**–**d** (5 µM) toward various G4 oligonucleotides.

G4 Oligonucleotides	1a ∆T_m_ (°C)	1b ∆T_m_ (°C)	1c ∆T_m_ (°C)	1d ∆T_m_ (°C)
FdxT	0.7	0.7	1.7	0.1
F25Cebt	14.8	13.8	17.5	14.9
FmycT	19.9	19.6	22.2	19.9
Fkit1T	16.8	18.9	15.6	17.1
F21T	19.7	18.9	23.5	19.1
F21CTAT	9.2	9.0	12.2	8.9
FTBAT	7.8	6.9	17.6	10.4
FBom17T	11.3	7.3	22.8	12.3
FPf1T	6.5	3.8	10.3	9.2
FPf8T	4.2	5.9	13.7	9.7

**Table 4:** In vitro sensitivity of compounds **1a**–**c** on two human myeloid leukaemia cell lines, and cytotoxicity on human peripheral blood mononuclear cells PBMNC + PHA.

Ligands	K562 IC_50_ (µM)	HL60 IC_50_ (µM)	Cytotoxicity on Activated Human Peripheral Blood Mononuclear Cells (PBMNC) PBMNC + PHA IC_50_ (µM)
**1a**	>50	>50	>50
**1b**	3.0 ± 0.5	18 ± 2.0	5 ± 0.3
**1c**	>50	>50	>50

**Table 5:** List of DNA sequences used in the FRET assay.

Sequence Name	Sequence(5′–3′)
ds26	CAATCGGATCGAATTCGATCCGATTG
F21T	Fam-G_3_TTAG_3_TTAG_3_TTAG_3_-Tamra
Fbom17T	Fam-G_2_TTAG_2_TTAG_2_TTG_2_-Tamra
FdxT	Fam-TATAGCTAT-hexaethyleneglycol-TATAGCTATA-Tamra
FmycT	Fam-TTGAG_3_TG_3_TAG_3_TG_3_TAA-Tamra
F25cebT	Fam-AG_3_TG_3_TGTAAGTGTG_3_TG_3_T-Tamra
FtbaT	Fam-G_2_TTG_2_TGTG_2_TTG-Tamra
c-myc	TTGAG_3_TG_3_TAG_3_TG_3_TAA
22AG	AG_3_TTAG_3_TTAG_3_TTAG_3_
24TTG	TTGGGTTAGGGTTAGGGTTAGGGA
22CTA	AGGGCTAGGGCTAGGGCTAGGG

## Supplementary Materials

The following are available online at www.mdpi.com/1420-3049/23/1/81/s1, Figure S1: Normalized melting curves obtained for ligands (a) 1a, (b) 1b, (c) 1c, (d) 1d, (e) 1e and (f) 1f with F21T (0.2 µM) in K^+^ conditions, Figure S2: Normalized melting curves obtained for ligands (a) 1a, (b) 1b, (c) 1c and (d) 1d (with F21T (0.2 µM) in Na^+^ conditions.
