# Supplementary material for: Design, Synthesis and Biological Evaluation of New Substituted Diquinolinyl-Pyridine Ligands as Anticancer Agents by Targeting G-Quadruplex
Source: Molecules. 2017 Dec 30;23(1):81. doi: 10.3390/molecules23010081 (PMC6017375; doi:10.3390/molecules23010081)
Supplement: Supplementary file 1 [file molecules-23-00081-s001.pdf]

SUPPORTING INFORMATION

Design, Synthesis and Biological Evaluation of New Substituted Diquinolinyl-pyridine Ligands as Anticancer Agents by Targeting G-quadruplex

Rabindra Nath Das[1], Edith Chevret[2], Vanessa Desplat[3], Sandra Rubio[1], Jean-Louis Mergny[1,4],* and Jean Guillon[1]*

1 Univ. Bordeaux, ARNA laboratory, INSERM U1212, UMR CNRS 5320, UFR des Sciences Pharmaceutiques, 33076 Bordeaux cedex, France; rabindra.das@u-bordeaux.fr
2 Univ. Bordeaux, INSERM U1053, Cutaneous Lymphoma Oncogenesis Team, 33076 Bordeaux cedex, France
3 Univ. Bordeaux, INSERM U1035, Cellules souches hématopoïétiques normales et leucémiques, UFR des Sciences Pharmaceutiques, 33076 Bordeaux cedex, France
4 Institute of Biophysics of the CAS, v.v.i., Kralovopolska 135, 612 65 Brno, Czech Republic

* Correspondence: jean-louis.mergny@inserm.fr; jean.guillon@u-bordeaux.fr; Tel.: +33557571652

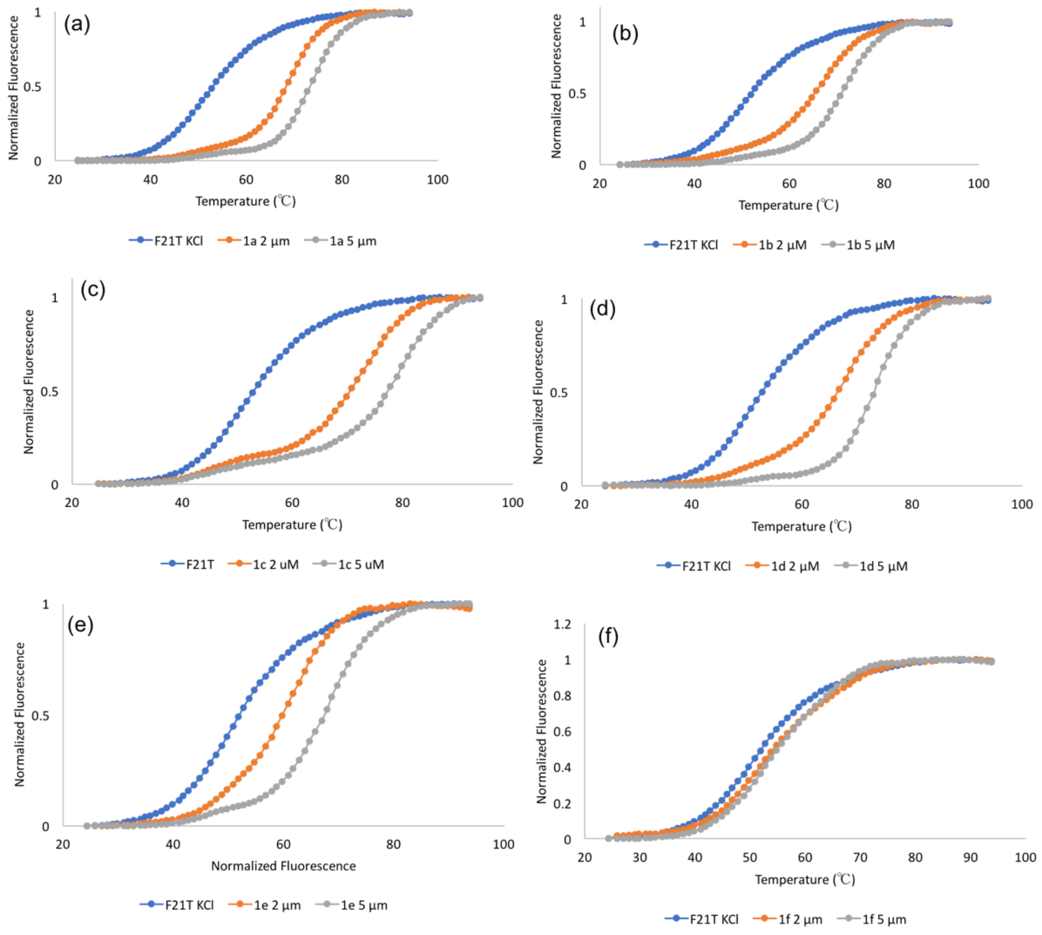

Figure S1. Normalized melting curves obtained for ligands (a) 1a, (b) 1b, (c) 1c, (d) 1d, (e) 1e and (f) 1f with F21T (0.2 µM) in K^+ conditions.

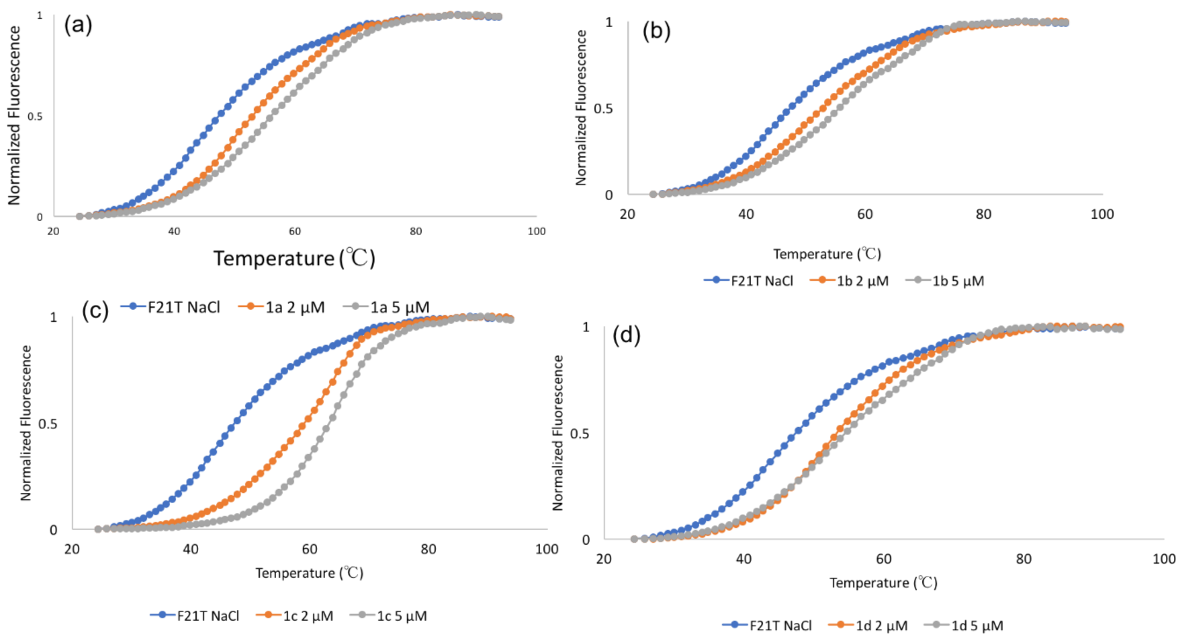

Figure S2. Normalized melting curves obtained for ligands (a) 1a, (b) 1b, (c) 1c and (d) 1d (with F21T (0.2 μM) in Na^{+} conditions.
